# Relationships Between Performance and Injury Occurrence in Athletics (Track and Field): A Pilot Study on 8 National-Level Athletes From Sprints, Jumps and Combined Events Followed During at Least Five Consecutive Seasons

**DOI:** 10.3389/fspor.2022.852062

**Published:** 2022-05-04

**Authors:** Joris Chapon, Laurent Navarro, Pascal Edouard

**Affiliations:** ^1^Inter-university Laboratory of Human Movement Sciences (LIBM EA 7424), University of Lyon, University Jean Monnet, Saint-Étienne, France; ^2^Mines Saint-Etienne, INSERM, U 1059 Sainbiose, Centre CIS, Univ Lyon, Univ Jean Monnet, Saint-Étienne, France; ^3^Sports Medicine Unit, Department of Clinical and Exercise Physiology, University Hospital of Saint-Etienne, Faculty of Medicine, Saint-Étienne, France

**Keywords:** health protection, sports injury prevention, injury risk, sports rehabilitation, athletics and championships

## Abstract

**Background:**

Performance success or failure in athletics (Track and Field) and the capacity to succeed are driven at the adult level, like in other sports, by many factors, injury being one of them. More information regarding the potential relationships between performance and injuries in athletics is needed.

**Objective:**

To analyse the potential association between performance and occurrence of injuries in national-level athletics athletes from sprints, jumps and combined events through several seasons.

**Methods:**

We performed a retrospective analysis of performance and injury data collected prospectively in 8 national-level athletics athletes followed during at least five consecutive seasons from 2009 to 2019. For each athlete, injuries data [total injuries (injuries) and time-loss injuries (TLI)] were collected by the same sports medicine physician throughout the study period using a medical attention injury definition. Performances during official competitions were collected on the French Federation of Athletics website, and included (i) any participation in national championships, (ii) any participation in an international competition (i.e., being national team member for an international competition), (iii) any podium at the national championships, (iv) any podium at an international competition, and (v) performance metrics normalised to the world record (WR) of the respective athletics speciality (%WR). For each athlete, we performed a descriptive analysis of the performances and injuries. We also performed four binomial logistic regressions with (1) national championships participation (yes/no) or (2) international competition participation (yes/no) as dependent variables, and injuries (yes/no) or TLI (yes/no) as independent variables, adjusted for individual athlete and number of seasons, and in models on participation in international competitions, was also adjusted for national championship participation (yes/no), with Odd Ratios (OR) with 95% confidence intervals (95%CI).

**Results:**

Among the 8 national-level athletics athletes included in the present study, cumulated 155 injuries, including 52 TLI (33.5%). There was an average of 2.7 ± 1.7 injuries and 0.9 ± 0.6 TLI per athlete per season over the study period. The occurrence of injuries was significantly associated with higher odds of national championships participation (OR = 4.85 [95% CI 3.10 to 3050.5], *p* = 0.021). The occurrence of TLI was significantly associated with higher odds of national championships participation (OR = 133.6 [95% CI 4.92 to 14251.5], *p* = 0.013). The occurrence of injuries or TLI were associated with insignificantly lower odds of international championships participation.

**Conclusions:**

Our present pilot study confirms that injuries are part of an athletes' life. The occurrence of at least one injury was associated with higher odds of participation in a national championship, whereas the absence of at least one injury was associated with higher odds of participation in an international championship. We hypothesised that the length of the season can play a role in the risk of injury occurrence, but if the athlete wants to reach his/her highest level, decreasing the risk of injuries seems to be of importance. Despite the caution that should be taken in the interpretation of our results, our present study confirms the interest and relevance of injury risk reduction approach in athletics.

## Introduction

Performance success or failure in athletics (Track and Field) and the ability to succeed at the adult level are driven, such as in other sports, by many factors (Huxley et al., 2014), injury being one of them. Recent articles, mostly regarding team sports such as football, basketball, or rugby (Drew et al., 2017), have reported a relationship between performance success or failure and injuries. In the Australian Football League, the availability of football players was associated with a higher position table of the teams, meaning that if players were not available due to injuries the rankings of the teams were lower (Hoffman et al., 2020). In basketball, the number of games missed due to an injury or illness have been reported to be associated with lower percentage of wins (Podlog et al., 2015). It is now admitted that less you are injured more you can practice or compete and less your team tends to lose (Drew et al., 2017). But team performance is an association of individual players, and these conclusions cannot be fully extrapolated to one specific athlete in an individual sport.

In athletics, few articles have analysed the potential relationships between performance and injuries (Raysmith and Drew, 2016; Edouard et al., 2019, 2021). During eight international athletics championships, Edouard et al. (2019) reported that lower numbers of injuries per registered athlete were correlated with higher number of medals and gold medals per registered athletes, when analysing country participation grouped according to country team sizes. Edouard et al. (2021) reported that being injured during international combined event competitions was associated with lower odds of winning a medal during the respective competition. To our knowledge, only Raysmith and Drew (2016) reported results on the relationships between performances and injuries during an athletics season follow-up. They reported that injuries occurring during the 6-months preparation period of an international competition, and related loss in training time, have a negative effect on the performance success for the respective competition in international-level athletes. All these results support the hypothesis that injuries lead to negative consequences on the performance in athletics. However, this needs to be confirmed with new studies.

In this context, the primary aim of our study was to analyse the potential association between performance and occurrence of injuries in national-level athletics athletes from sprints, jumps and combined events through several seasons. We chose to focus our study on (i) national-level athletes because the number of competitions per season is often greater than for lower-level athletes since they have the opportunity to participate in more competitions, and such athletes are often those who invest more time and energy in athletics practice, (ii) athletes from explosive athletics disciplines (i.e., sprints, jumps and combined events) because their injuries are expected to present similar characteristics making the sample more homogeneous (Edouard et al., 2015, 2020), and (iii) a period longer than one season to allow a more representative view of athletes at the individual level.

## Methods

### Study Design and Procedure

We performed a retrospective analysis of performance and injury data collected prospectively in a cohort of national-level athletics athletes during at least five consecutive seasons between 2009 and 2019. The study was conducted in accordance with the Helsinki Declaration and was reviewed and approved by the Saint-Etienne University Hospital Ethical Committee (Institutional Review Board: IORG0007394).

### Population

The population was selected from the athletes licensed at the French Federation of Athletics for competition within the Coquelicot 42 Club (http://coquelicot42.athle.com) practicing sprints, jumps and combined events, and among athletes who were members of the Elite or Training Centre groups between 2009 and 2019 (*n* = 132).

To be included in the present study, athletes must have met the following inclusion criteria: (i) athletes licensed at the French Federation of Athletics for competition, (ii) licensed within the Coquelicot 42 Club, (iii) practicing sprints, jumps or combined events, (iv) competing at least at the national level, (v) being member of the Coquelicot 42 Elite or Training centre groups, (vi) being medically followed in the Sports Medicine unit of the University Hospital of Saint-Etienne (https://www.chu-st-etienne.fr), and (vii) competing during at least five consecutive seasons. Athletes in the Elite group were those from the Coquelicot 42 Club ranked in the national top 30 all categories (i.e., combining all age categories together), and athletes in the Training Centre group were those ranked in the national top 30 of his or her category, in their main athletics speciality. These athletes had thus a minimum of a national level to be included in the groups and thus in the study. The club of Coquelicot 42 had a medical convention with the University Hospital of Saint-Etienne (https://www.chu-st-etienne.fr) allowing a privileged medical follow-up of the athletes' member of these two groups by the same sports medicine physician (PE).

Among the 132 different athletics athletes who belonged to the Training and Elite groups from 2009 to 2019, ten athletes (8%) have belonged to these groups for at least five consecutive years and eight (6%) athletes met all the inclusion criteria and were thus included in the present study.

### Athletes' Characteristics Data Collection

Athletes' characteristics were collected in the medical files of the University Hospital of Saint-Etienne at the start of the follow-up: sex, date of birth, main athletics speciality. Date of birth was collected to calculated age of each included athlete at the different occurrences of performances or injuries. The main athletics speciality corresponded to that declared on the FFA website; if nothing was declared we chose the speciality in which the athlete performed at the highest level during his/her career, calculated by the percentage of performance in comparison to the World Record (see below).

### Performance Definition and Data Collection

The performance corresponded to an athletics performance performed during an official athletics competition held by the FFA or other national or international athletics federations. These performance data were retrospectively collected on the FFA database for each athlete included (https://www.athle.fr/asp.net/main.html/html.aspx?htmlid=5268). For our present study, we only considered for analysis the performances of the main speciality of the included athletes during the period of follow-up.

Regarding athletics performance, we first collected for each included athlete (i) any participation in national championships, (ii) any participation in an international competition (i.e., being national team member for an international competition), (iii) any podium at the national championships, and (iv) any podium at an international competition. These performances were named in the present study as *prize list*. The date of each performance occurrence was also collected. For the primary analysis, we only considered (i) any participation in a national championship, and (ii) any participation in an international competition. Indeed, participation at a national or international competition represents the global result of a season for an athlete, and often the goal of the season as reported by Raysmith and Drew (2016). On the contrary, a podium or a victory is the result of a single competition where several “parasitic” factors can play a role on the performance, such as for instance number of participants, climatic conditions, stress, etc., all of which are diluted by the number of competitions when considering annual participation in national or international competitions. Our choice was therefore to consider the most representative outcomes of the entire season and not an isolated point.

We then collected the athletics performances in time (in seconds) for sprints, distance (in meters) for jumps, and total of points for combined events. The date of each performance occurrence for %*WR* was also collected. All the collected data were then normalised to the World Record (WR) of the respective sex and speciality at the time of the performance. This performance was named *%WR* in the present study.

### Injury Definition and Data Collection

Based on the definitions from the athletics consensus statement (Timpka et al., 2014) and the International Olympic Committee consensus statement (Bahr et al., 2020), we chose to use in our study the definition of injury as “a physical complaint or observable damage to body tissue produced by the transfer of energy experienced or sustained by an athlete during participation in athletics training or competition, that received medical attention, and regardless of its consequences with respect to impairments in connection with competition or training.” For the injury data analysis, we used two states of injury: “*total injuries*” corresponding to total injuries reported by athletes to the medical staff and recorded by the medical staff during the period of follow-up, and then named “*injuries*”; and “*Time Loss Injury*” (TLI) corresponding to injuries resulting in suspending the athlete's practice.

Injury data was prospectively collected for all included athletes in medical files by the same sports medicine physician (PE). For each injury with medical attention, the athletes included in study contacted the doctor. Each injury that received medical attention was characterised during the consultation and a note was written in medical file with the details of the consultation. Remote monitoring could have been done before and/or after the consultation by email or by phone. For each athlete, injury data were collected retrospectively in medical files and centralized in an excel file, and then anonymised for analysis. Each injury was characterised by (1) injury location: lower limb, upper limb, trunk, thorax, and multi (when more than one area was involved); and (2) injury type: muscle, joint, tendon, bone, ligament, skin and multi (when more than one tissue was involved).

### Statistical Analysis

We first performed a descriptive analysis of the collected data, using numbers with percentages for categorical variables, and means with standard deviations (± SD) for continuous variables. We calculated the number of national championship participations, international competition participations, national podiums, international podiums, total of *prize list*, %WR, *injuries* and *TLI* per athlete over the study period and per season. For each included athlete, we carried out descriptive analysis of both the evolution of *%WR* and the occurrence of *injuries* and *TLI* over time in relation to athlete age.

For the primary analysis, we determined for each athlete and each season if there was at least one national championship participation (yes/no), international competition participation (yes/no), injury (yes/no), or TLI (yes/no). To analyse the potential association between performance and occurrences of injuries (i.e., primary analysis), we performed four binomial logistic regressions with participation in either national or international championships as the dependant variables, and either injuries or TLI as independent variables. Logistic regressions of participation in national championships were adjusted to individual athletes and number of seasons. Logistic regressions of participation in international championships were adjusted to individual athletes and number of seasons and participation in national championships. Risk indicators were presented as Odd Ratios (OR) with 95% confidence intervals (95%CI). The significance level was initially set at *P* < 0.05.

Data analysis was performed using excel (©2021 Microsoft Corporation), R [version 4.0.2, © Copyright 2020 The Foundation for Statistical Computing (Comprehensive R Archive Network, http://www.R-project.org)] using R library “questionr,” and Python (version 3.8.0© Copyright 2001–2021 Software Foundation).

## Results

### Population

The characteristics of the eight included athletes are presented in the Table 1. There were 3 women (37.5%) and 5 men (62.5%), corresponding to 1 hurdler (12.5%), 3 jumpers (37.5%) and 4 combined events athletes (50.0%). The average age of the 8 athletes at their inclusion in the study was 19.8 ± 4.9 years. Together, the eight follow-ups represented a total of 67 seasons of follow-up, with an average of 8.4 ± 2.3 seasons per athlete.

**Table 1:** Characteristics of the included athletes with the information on competitions, prizes won, performance expressed as percentages of the World Record (%WR) and on injuries with injuries and time loss injuries (TLI).

**Athletes**	**Sex**	**Main athletics speciality**	**Age at start of follow-up**	**Number of seasons of follow-up**	**Number of prize list**	**Number of national championships participations**	**Number of national podiums (medals)**	**Number of international championships participations**	**Number of performances as %WR**	**Maximum of %WR**	**Number of Total injuries**	**Number of TLI**	**Number of prize list per season**	**Total injuries per season**	**TLI per season**
1	M	Combined Event	14.4	10	21	15	4	2	44	0.81	9	0	1.7	0.9	0
2	M	Combined Event	18.6	10	21	21	0	0	45	0.78	16	6	2.1	1.6	0.6
3	F	Combined Event	21.5	9	43	35	8	0	63	0.79	23	6	3.9	2.6	0.7
4	M	High Jump	13.2	12	47	33	10	4	167	0.90	18	7	2.8	1.5	0.6
5	F	400 m hurdles	19.5	7	10	8	1	1	27	0.81	20	7	1.1	2.9	1
6	M	Long Jump	24.7	8	21	17	3	1	127	0.76	15	6	2.1	1.9	0.8
7	M	Combined Event	18.4	6	16	16	0	0	41	0.79	24	10	2.7	4	1.7
8	F	Pole Vault	27.9	5	33	20	9	4	77	0.84	30	10	4	6	2
**Total**	**-**	**-**	**-**	**67**	**212**	**165**	**35**	**12**	**591**	**-**	**155**	**52**	**-**	**-**	**-**
**Mean**	**-**	**-**	**19.8**	**8.4**	**26.5**	**20.6**	**4.4**	**1.5**	**73.9**	**0.81**	**19.4**	**6.5**	**2.6**	**2.7**	**0.9**
SD	-	-	4.9	2.3	13.1	9.1	4.1	1.7	48.7	0.04	6.4	3.1	1.0	1.7	0.6

*M, male athlete; F, female athlete; SD, Standard Deviation*.

### Performance

Athlete's individual performance, i.e., number of prizes and number of prizes per season, %WR values and maximum %WR, are reported in Table 1. Regarding the prizes lists, all athletes were selected for at least one national championship, with an average of 20.6 ± 9.1 participations in national championships (Table 1).

### Injuries

A total of 155 injuries was collected, including 52 TLI (33.5%). There was an average of 2.7 ± 1.7 injuries and 0.9 ± 0.6 TLI per athlete per season over the study period (Table 1). The main injury location was the lower limb (85%) and the main type was muscle (44%).

### Primary Analysis: Association Between Performance and Occurrence of Injuries

Regarding our primary aim, the results of the four binomial logistic regressions are presented in the Table 2. The occurrence of injuries was significantly associated with higher odds of national championships participation (OR = 4.85 [95% CI 3.10 to 3050.5], *p* = 0.021) (Table 2). The occurrence of TLI was significantly associated with higher odds of national championships participation (OR = 133.6 [95% CI 4.92 to 14251.5], *p* = 0.013) (Table 2). Participation in international competitions tended to coincide with lower occurrences of injuries and of TLI, but this association was not significant (Table 2).

**Table 2:** Odds ratio (OR) with 95% confidence interval (95% CI) of the association of injury occurrence [injuries or time-loss injuries (TLI) (yes/no)] with performance (1) participation in national championships (yes/no) or (2) participation in international championships (yes/no) adjusted for individual athlete and number of season (adjustment variables) and for participation in national championships for the participation in international championships, using a logistic regression.

**Outcomes:**	**OR**	**95%CI**	***p*** **value**
Participation in national championships			
Injuries	4.85	(3.10 to 3050.5)	0.021
Participation in national championships			
Time-loss injuries	133.6	(4.92 to 14251.5)	0.013
Participation in international championships			
Injuries	0.14	(0.01 to 1.30)	0.096
Participation in national championships	1.91	(1.02 to 915.0)	0.075
Participation in international championships			
Time-loss injuries	0.19	(0.01 to 2.26)	0.225
Participation in national championships	9.84	(0.68 to 375.2)	0.128

## Discussion

The main findings of the present study were that (1) the occurrence of at least one injury (or TLI) was associated with higher odds of participation in a national championship, (2) the absence of occurrence of at least one injury (or TLI) was associated with higher odds of participation in an international championship, (3) for athletes with a period of increased performance, the descriptive analysis reported no TLI during this period (e.g., athletes 1 and 4), and (4) the descriptive analysis confirms that injuries are part of athletes' life. Given the small number of included athletes and the limitations described below, the present study should be seen as a pilot study and the results should be taken with caution.

### Association Between Performances and Injuries

The results of our primary analysis (Table 2) show that the occurrence of injury (injuries and TLI) is associated with higher odds of participation in a national championship. These results may at first glance seem counterintuitive and do not support the conclusions of Raysmith and Drew (2016). Indeed, national championships are a key objective of an athlete's season, and it seem logical to think that the absence of injury would increase the chance of participation in national championships. However, an athletics season in France is long, and the national championships are often the last competitions of the season. Thus, an athlete who participates in national championships will have a long(er) season, and consequently increase his/her exposition to the risk of injury occurrence. An athlete who does not participate in the national championships will have a short(er) season, a potentially lower number of competitions than an athlete who participates in the national championships, and consequently will decrease his/her exposition to the risk of injury occurrence. We thus hypothesis that our present results could be the consequence of the season's length. This hypothesis could have direct practical applications by enabling athletes to anticipate season length or the number of competitions they wish to participate in.

Conversely, our analysis regarding the participation in an international championship showed absence of injury during the season was insignificantly associated with higher participation in an international championship (Table 2). Although the results were not significant, these results are interesting and in agreement with those from Raysmith and Drew (2016). Suffering an injury during a season seems more detrimental when aiming at participating in an international championship than in a national championship. In our population, on the total follow-up, there were on average 20.6 ± 9.1 in national championships for only 1.5 ± 1.7 in international championships. We could interpret these results as implying that it is “easy,” to compete in national championships for the athletes included in our population given their level of practice: whatever their health status during the season they can (in majority) be selected for national competition. However, participation in an international championship is more complex and may require a very good health status. Despite these interesting results, one must not lose sight the limitations of our study.

**Figure 1:**
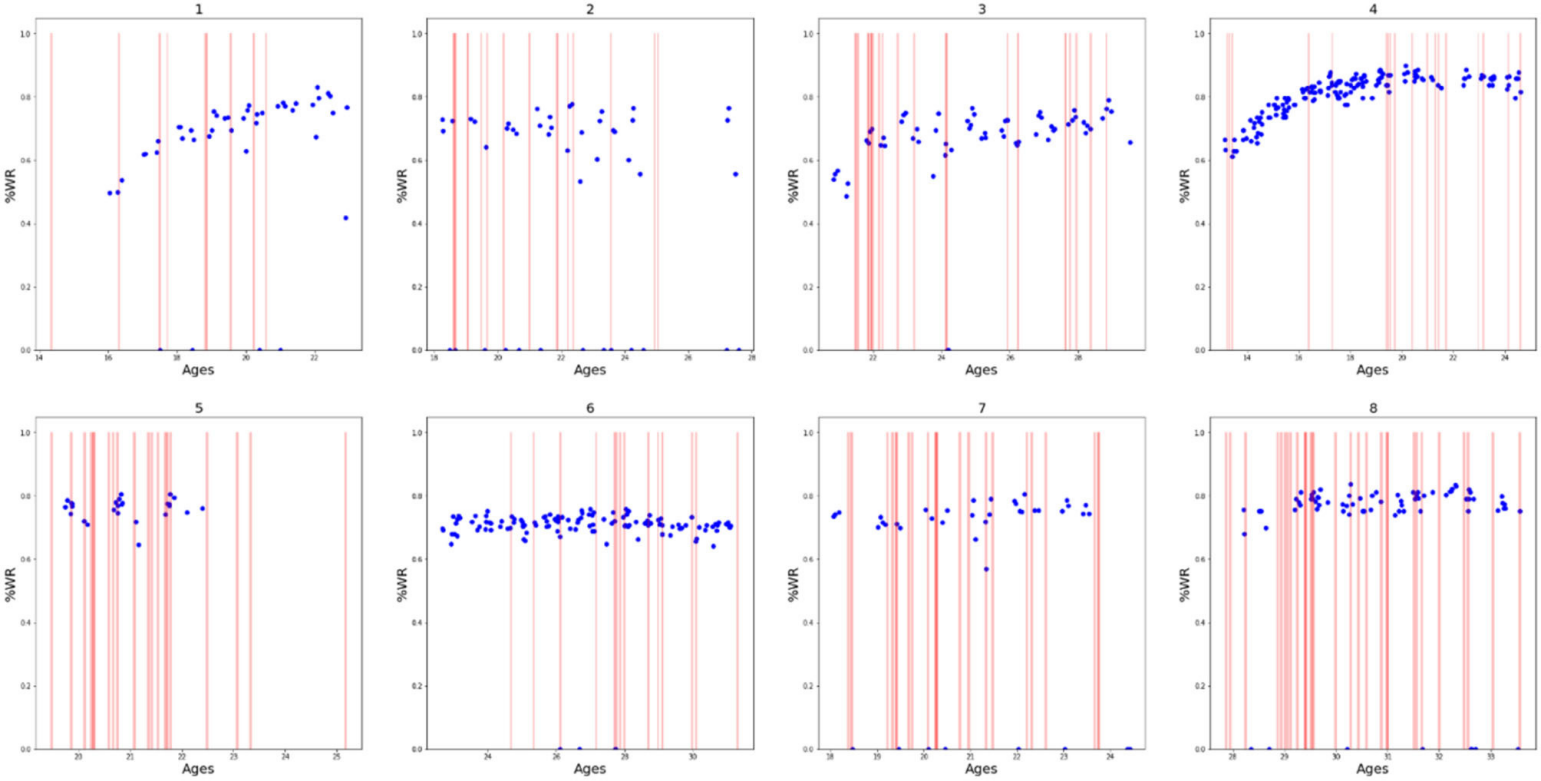
Evolution of the performances (represented by the percentage of the World Record, in blue dots) for each included athlete over time (represented by the athlete's age) with occurrence of injuries (in red vertical line). The meaning of numbers (1–8) are the number of each athlete of the study.

**Figure 2:**
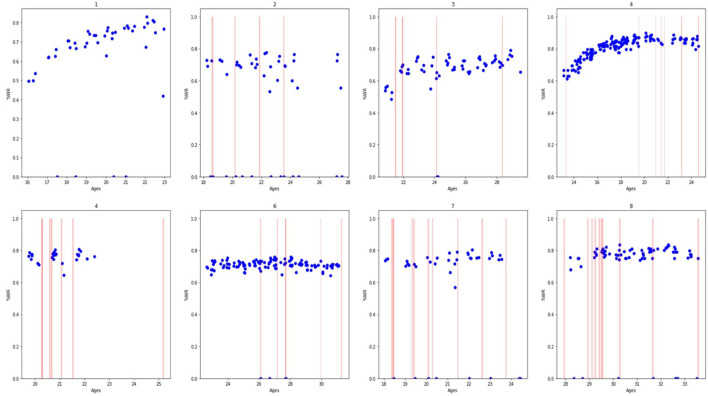
Evolution of the performances (represented by the percentage of the World Record, in blue dots) for each included athlete over time (represented by the athlete's age) with occurrence of time loss injuries (in red vertical line). The meaning of numbers (1–8) are the number of each athlete of the study.

In Figures 1, 2, we present a descriptive analysis of the evolution of athlete performances represented by the %WR in connection with the occurrence injuries and TLI, respectively. All athletes had at least one injury, confirming that injuries are part of the athletes' life. It seems that the two athletes with the most important and visible increase in performances (i.e., athletes 1 and 4) were those with the lowest “density” of injuries in Figure 1 and TLI in Figure 2, and the lowest number of injuries and TLI per season (Table 1). We can also note in Figure 2 that there were no TLI during the rising phases of %WR for athletes 1 and 4. Such visual descriptive analysis seems to confirm that no or less injuries favour improved performances.

### Performance Evolution and Injuries

Our present study reports the individual athlete's evolution of performances according to age (Figures 1, 2). The included athletes had heterogeneous age during the follow-up; the different athletes were not included at the same period of their career; some were at the beginning (e.g., athlete 4), others were at the end (e.g., athlete 8), and others had their entire career recorded (e.g., athletes 1 and 4). This could explain the difference between the individual evolution of %WR. We can see a positive change in performances for athletes who were included in their youth (athletes 1 and 4). For the athletes included at an older age the positive evolution was less important or even a plateau and a regression. The important positive evolution phase can be seen in younger categories, and around 20 years of age progression becomes very limited. Boccia et al. (2017) concluded that the “most of the successful early-on, under 18 years world-class jumpers did not manage to maintain the same level of competitiveness in adulthood since they experienced a plateau in performance from 20 years of age.” This inability to maintain their progress after 20 years of age can be explain by different factors, of which injury may be an important one.

For instance, for athlete 4 (an international high jump athlete), we can see his positive evolution in percentage of world record with the occurrence of injuries. This evolution is similar to that described by Boccia et al. (2017) and we reported the same evolution with time and age. We can see a progression from age 13 to his twenties followed by a plateau. In our present study, the addition of injuries occurrence in Figures 1, 2 can help better understand the evolution of performances, by presenting one of the multiple aspects that can impact performance. In our hypothesis, injury occurrence can be a factor that limits the positive evolution of performances. For athlete 4, we can hypothesis that injuries occurring at the start of this athletes' performance plateau had negative effect on his positive evolution in performance, as the occurrence of injuries was more frequent in the performance plateau than during the positive evolution phase. This difference of occurrences raises the question of an association between performance and injury occurrence. Of course, it is important to keep in mind that performance and injury are multifactorial and just injuries cannot explain all variations in performance. As we saw above this athlete was not injured during his positive evolution. This absence of injuries can be explained by the fact that the level of his performance was not excessively high, so presumably his body was able to undergo this level of demand. Injuries appear after athletes performed at their top performance. These injuries may have come about because the demand on the athletes was too high for their bodies to handle, and/or perhaps injuries are what stopped his improvement in performance. These are only hypotheses as causal relationships cannot be drawn from the present study.

### Limitations

The number of athletes included in the present study could be considered as small. Also we used a medical attention definition (Timpka et al., 2014; Bahr et al., 2020). Thus, some injuries or injury complaints may have been missed. All athletes had different sensitivities and could have contacted medical staff at different levels of pain (Kakiashvili et al., 2016), therefore injuries recorded with medical attention may have had been more or less severe. We chose this approach since we think that injuries with a substantial negative impact on athletic practice, and thus important in the context of our present study aim, would force athletes to see the sports physician. In addition, our approach can allow for greater precision of injury diagnosis. Retrospective data collection from medical files is based on information that has been reported prospectively. The duration of the time loss or the training adaptation was difficult to determine based solely on the information in the medical files and without access to the training data. This limitation induced a difficulty to classify TLI, and some TLI could have been missed. Using competition performances also lead to limitations, since the performance values depend on the date of the competition and several factors independent of injuries can contribute to competition occurrence and performance (e.g., official calendar, athlete's selection, athlete's season strategy, weather). The competition outcome may be a good indicator of the athlete's level, but is influenced by many different factors such as psychological or external factors independent of athlete's level, the competition performance is not only determined by the health status (injured or not). Another limitation on competition performance is the competition planning. Athletics is not similar to team sports, there are no regular or weekly matches throughout the season. In athletics, seasons are split in winter and summer seasons with long periods without competition. For periods without competitions there are no performance data, so it is difficult to analyse relationships between performance and injury occurrence during these periods based on the competition performances only. In addition, most injuries occurred during periods of active training (Edouard et al., 2015), making it difficult to link injuries with competition outcomes later in the season. The absence of training data cannot be replaced simply by interpreting the irregular competition performance data. Indeed, regular monitoring of performance in training associated with training load data would be relevant. Exposure to the risk (training and competition duration and intensity) was not collected. Nevertheless, evolution of performance data has provided some new evidence and raised new questions. Given these limitations our present study should be considered as a pilot study.

### Strengths

Despite these limitations, this study followed athletes during a long period of time and not only few times before and/or during a competition. Indeed, except Raysmith and Drew (2016), there are no other studies that followed a group of athletes as long as in our study. Some athletes included in our study were followed at least 10 years. The included athletes were high-level athletes: five athletes have been selected in French national team at least one time. This present preliminary study brings additional information on the association between performance and injuries. All of the above limitations are areas for improvement in future studies.

### Perspectives

This present study should be considered as a pilot study, but understanding gained from this study can help us to design future studies that can help to enhance improvements of our knowledge of the relationships between performance and injuries in athletics. Such studies should include a prospective design, with prospective monitoring of training including the reasons of absence from training, regular monitoring of physical performances and health, and include, if possible, the monitoring of other factors that may affect performance, such as fatigue, sleep, or psychological factors.

## Conclusions

The main findings of our pilot study were that the occurrence of at least one injury was associated with higher odds of participation in a national championship, but the absence of occurrence of at least one injury was associated with higher odds of participation in an international championship. We hypothesised that the length of the season can play a role in the risk of injury occurrence between these two levels of competition, but if the athlete wants to reach his/her highest level, decreasing the risk of injuries seems to be of importance.

## Data Availability Statement

The raw data supporting the conclusions of this article will be made available by the authors, without undue reservation.

## Ethics Statement

The studies involving human participants were reviewed and approved by the Saint-Etienne University Hospital Ethical Committee (Institutional Review Board: IORG0007394). Written informed consent to participate in this study was provided by the participants' legal guardian/next of kin.

## Author Contributions

All authors participated to the manuscript and approved its submission.

## Conflict of Interest

The authors declare that the research was conducted in the absence of any commercial or financial relationships that could be construed as a potential conflict of interest.

## Publisher's Note

All claims expressed in this article are solely those of the authors and do not necessarily represent those of their affiliated organizations, or those of the publisher, the editors and the reviewers. Any product that may be evaluated in this article, or claim that may be made by its manufacturer, is not guaranteed or endorsed by the publisher.
